# Clinical Trial Readiness in Limb Girdle Muscular Dystrophy R1 (LGMDR1): A GRASP Consortium Study

**DOI:** 10.1002/acn3.70049

**Published:** 2025-04-16

**Authors:** Stephanie M. Hunn, Lindsay N. Alfano, Aileen Jones, Amanda Butler, Linda P. Lowes, Megan A. Iammarino, Natalie F. Reash, Lindsay Pietruszewski, Sandhya Sasidharan, Melissa Currence, Jeffrey M. Statland, Talia Strahler, Robert Will, Matthew Wicklund, Stacy Dixon, Renee Augsburger, Tahseen Mozaffar, Katie M. Laubscher, Shelley R. H. Mockler, Katherine D. Mathews, Nikia Stinson, Doris G. Leung, Molly M. Stark, Rebecca A. Horton, Peter B. Kang, Meredith K. James, Amanda Clause, Conrad C. Weihl, Nicholas E. Johnson, GRASP‐LGMD Consortium

**Affiliations:** ^1^ Washington University School of Medicine St. Louis Missouri USA; ^2^ The Abigail Wexner Research Institute at Nationwide Children's Hospital Columbus Ohio USA; ^3^ The Ohio State University College of Medicine Columbus Ohio USA; ^4^ Virginia Commonwealth University Richmond Virginia USA; ^5^ University of Kansas Medical Center Kansas City Kansas USA; ^6^ University of Colorado Aurora Colorado USA; ^7^ University of California Irvine California USA; ^8^ Center for Disabilities and Development, University of Iowa Health Care Stead Family Children's Hospital Iowa City Iowa USA; ^9^ Kennedy Krieger Institute Baltimore Maryland USA; ^10^ Greg Marzolf Jr. Muscular Dystrophy Center and Department of Neurology University of Minnesota Medical School Minneapolis Minnesota USA; ^11^ The John Walton Muscular Dystrophy Research Centre, Newcastle University and Newcastle Hospitals NHS Foundation Trust Newcastle Upon Tyne UK

**Keywords:** limb girdle muscular dystrophy, natural history, outcome measures

## Abstract

**Objective:**

Identifying functional measures that are both valid and reliable in the limb girdle muscular dystrophy (LGMD) population is critical for quantifying the level of functional impairment related to disease progression in order to establish clinical trial readiness in the context of anticipated therapeutic trials.

**Methods:**

Through the Genetic Resolution and Assessments Solving Phenotypes in LGMD (GRASP‐LGMD) Consortium, 42 subjects with LGMDR1 were enrolled in a 12‐month natural history study across 11 international sites. Each subject completed a battery of clinical outcome assessments (COA), including the North Star Assessment for Limb Girdle‐Type Dystrophies (NSAD), 10‐m walk/run, and Performance of the Upper Limb (PUL), in addition to several patient‐reported outcome measures (PROM).

**Results:**

In this baseline cross‐sectional analysis, significant correlations were found between COAs and PROMs, with significant differences in the performance of assessments based on subjects' ambulatory status and genetic variant classification.

**Interpretation:**

The study has determined that the NSAD and other assessments are valid and reliable measures for quantifying the level of disease impairment in individuals with LGMDR1.

**Abbreviations**
10 m: 10‐Meter Walk/Run
100 m: 100‐Meter Timed Test
4SC: 4‐Stair Climb
9HPT: 9‐Hole Peg Test
COA: clinical outcome assessments
DASH: Disability of the Arm, Shoulder and Hand
FVC: Forced Vital Capacity
GRASP‐LGMD: The Genetic Resolution and Assessments Solving Phenotypes in LGMD
LGMD: limb girdle muscular dystrophy
NSAD: North Star Assessment for Limb Girdle‐Type Muscular Dystrophies
PROM: Patient‐Reported Outcome Measures
PROMIS‐57: Patient‐Reported Outcomes Measurement Information System
PUL: Performance of the Upper Limb
RFF: Rise From Floor
TUG: Timed Up and Go

## 1 Introduction

The limb girdle muscular dystrophies (LGMDs) are slowly progressive muscle diseases resulting in shoulder and pelvic girdle weakness due to pathogenic variants in a specific gene. Subtypes of LGMD are classified by each gene along with their inheritance pattern. There are 32 genes that have been found to cause LGMD, 27 of which have a recessive inheritance pattern compared to a dominant pattern [1, 2]. The clinical presentations of various LGMD subtypes are heterogeneous, with more severe subtypes, such as the sarcoglycanopathies, involving cardiac and respiratory muscles. Disease progression can be slow and highly variable, even within each subtype; therefore, establishing valid sensitive outcome measures has proved challenging. Phenotypic variability between subtypes contributes greatly to this challenge as there has yet to be one validated assessment that can universally be performed across all LGMD subtypes. Prior natural history studies have validated newly developed outcome measures in specific subtypes of LGMD, including the North Star Assessment for Limb Girdle‐Type Muscular Dystrophies (NSAD) in LGMDR2 [3, 4]. Demonstration of the validity and reliability of this assessment and others, as well as their ability to detect functional change over time, is needed in each LGMD subtype for therapeutic trial development.

Limb girdle muscular dystrophy type R1 (LGMDR1) is the most common subtype of autosomal recessive limb girdle muscular dystrophy in many populations. Pathogenic variants in the calpain 3 (*CAPN3*) gene lead to deficiency in the calpain‐3 protein or its activity in individuals with LGMDR1, resulting in progressive muscle weakness. The presentation of LGMDR1 is largely heterogeneous, with a wide range of symptom onset, including age of onset and identification of the first symptom, and a variable weakness progression pattern of upper versus lower extremities. Prior natural history studies have found symptom onset generally begins in the first or second decade of life, with an earlier age of onset typically leading to loss of ambulation at a younger age (24.5 years of age compared to 30.5 years of age) [5, 6]. Unlike other subtypes of LGMD that have less genotypic variability due to the prevalence of common founder mutations (i.e., LGMDR9) [7] or a paucity of pathogenic variants, such as LGMDD1, LGMDR1 has the greatest number of singleton pathogenic variants, leading to the question of whether or not this results in increased phenotypic variability.

A relationship between phenotype and specific pathogenic variants of the *CAPN3* gene has been reported, with individuals harboring missense variants presenting with milder phenotypes compared to those with null pathogenic variants who experience a more rapid loss in strength [8]. Null variants, resulting in a loss of calpain‐3 protein, often correspond with earlier symptom onset (11.6 years old for null variants compared to 16.6–17.5 years old for other variants) [8]. However, this correlation was based on a population of patients with limited heterogeneity, and whether this relationship holds across the full genetic spectrum of LGMDR1 has not yet been investigated. In comparison to other recessive LGMD subtypes, distal lower extremity muscles remain stronger in LGMDR1 [2]. Dysphagia and cardiopulmonary impairment are also less severe in LGMDR1 than in other subtypes of LGMD [2, 8].

The Genetic Resolution and Assessments Solving Phenotypes in LGMD (GRASP‐LGMD) consortium, consisting of 11 sites in the United States and 2 in Europe, aims to validate clinical outcome assessments (COA) across various types of LGMD. A lack of sensitive outcome measures continues to delay the implementation of therapeutic trials in LGMD, and with promising treatments on the horizon, there is an urgent need to validate disease‐specific assessments from natural history studies. The purpose of our study was to prospectively evaluate the feasibility, utility, and reliability of existing COAs in patients with LGMDR1.

## 2 Materials and Methods

As part of the GRASP Consortium, the GRASP‐01‐001 Defining Clinical Endpoints in LGMD study included enrollment of subjects with several types of LGMD divided into two study arms (NCT03981289) [9]. Here we report data from the baseline visits of patients with LGMDR1 (CAPN3) enrolled under Arm 1 of this larger trial. The study was overseen and approved by Western Institutional Review Board (WIRB), now known as WIRB–Copernicus Group (WCG IRB), and was active across a total of 11 sites with recruitment of subjects from local clinics, patient advocacy groups, and patient registries. Subjects provided written consent, accompanied by assent when appropriate. The study recruited subjects ages 4–65 years at the time of enrollment who were clinically affected (defined as weakness on bedside evaluation in a limb‐girdle pattern) with two genetically confirmed variants in the *CAPN3* gene to participate in this 12‐month longitudinal study. Our study excluded patients with a single *CAPN3* variant that could be consistent with autosomal dominant inheritance (LGMD D4). Subjects were also excluded from the study if pregnant, including a positive pregnancy test at any point during the trial, had a history of a bleeding disorder (platelet count < 50,000, current use of anticoagulant), or any other illness that would interfere with the ability to undergo safe testing or interpretation of the results of the study.

This study included a total of four visits over 1 year, with two back‐to‐back baseline visits, a 6‐month visit, and a 12‐month visit. Here we report on the baseline visits (Days 1 and 2) across the cohort. Subjects performed a battery of COAs at each visit, including the 10‐Meter Walk/Run (10 m), North Star Assessment for Limb Girdle‐Type Dystrophies (NSAD), Performance of the Upper Limb (PUL), 100‐Meter Timed Test (100 m), 4‐Stair Climb (4SC), Timed Up and Go (TUG), 9‐Hole Peg Test (9HPT), and spirometry [10, 11, 12, 13, 14, 15, 16]. These were administered by a trained clinical evaluator (physical or occupational therapist) who had completed training in the administration and scoring of the COA. Additionally, subjects completed patient‐reported outcome measures (PROM) at each time point, including the Activlim, Disability of the Arm, Shoulder, and Hand (DASH), and Patient‐Reported Outcomes Measurement Information System (PROMIS‐57) [17, 18, 19, 20]. In preparation for clinical trial readiness for LGMDR1, it is imperative to assess the test–retest reliability of selected COAs to ensure the assessments are a valid representation of the subject's true capabilities and are being performed consistently across multiple sites. Inter‐visit reliability can be calculated over a two‐day visit to quantify variability attributed to testing versus true disease progression. Due to the COVID‐19 pandemic and subsequent travel restrictions, the study protocol allowed for remote visits when feasible at approved sites.

Subjects were divided into two cohorts based on their performance on the 10 m at baseline [9]. Cohort A included subjects with a 10 m time of < 12 s at Baseline Day 1, and Cohort B included those with a 10 m time > 12 s or unable to safely ambulate without assistance, including the use of assistive devices. Cohort A completed the full battery of assessments at each visit, while the lower extremity functional assessments (100 m, 4SC, and TUG) were eliminated from Cohort B to focus on upper extremity functional abilities and reduce the overall burden of testing.

To explore possible genotype–phenotype correlations, subjects were also divided into three groups, “null,” “other,” and “null/other,” based on the expected effects of their CAPN3 variants on the encoded protein. The “null” variant category included nonsense variants, frameshift variants, an in‐frame deletion of exon 1, and variants expected to influence splicing, with one exception (the c.1746‐20C > G variant). The “other” variant category included missense variants, in‐frame deletions of one to several amino acids, and the c.1746‐20C > G variant. While this variant has been shown to influence splicing [21], we classified it as “other” since patients with this variant display variable deficits in calpain‐3 protein expression (ranging from absent to only mildly reduced), and homozygous carriers may not present with clinical disease.

Data analysis was performed using SPSS software version 28.0 (IBM SPSS, Chicago, IL, USA). Descriptive statistics were performed for the demographics of each cohort. Linear regression models were used to evaluate the performance of each functional assessment to determine the relationship between performance and time since symptoms onset. Unpaired *t*‐tests and Mann–Whitney tests were calculated to analyze the impact of disease duration, ambulatory status, and variant group on functional performance of COAs. Parametric (Pearson *R*) and non‐parametric (Spearman rho) correlation coefficients were also performed to assess potential relationships between the COAs, PROMs, and disease severity with Bonferroni correction to reduce the impact of bias across multiple comparisons. Intraclass correlation coefficient (ICC) and Bland–Altman plots were used to assess test–retest reliability of the COAs.

## 3 Results

### 3.1 Cohort Description

A total of 42 individuals with LGMDR1 enrolled in the study, with only one subject completing remote baseline assessments. The majority of LGMDR1 subjects were female (61.9%) with a mean age of 35.0 ± 15.7 years [range: 7–63 years] at the time of enrollment (Table 1). Subjects were categorized into one of three genetic variant groups depending on the expected effect of the variant on the calpain‐3 protein. Twelve of the subjects (28.6%) presented with *CAPN3* genetic variants classified as “null,” while 16 subjects (38.1%) had one “null” and one “other” *CAPN3* variant, and 14 subjects (33.3%) were found to have two “other” *CAPN3* variants. A complete cohort description can be found in Table 1.

**TABLE 1:** Demographics.

	*n*	Mean ± SD	Range	*p*
Age (years)	42	35.0 ± 15.7	7–63	ns
Cohort A	22	31.2 ± 16.2	7–58	
Cohort B	20	39.3 ± 14.3	15–63	
Null	12	31.4 ± 9.4	15–44	
Null/Other	16	32.1 ± 16.8	11–59	
Other	14	41.5 ± 17.5	7–63	
Symptom Onset (years)	42	16.7 ± 11.0	2–44	ns
Cohort A	22	19.6 ± 12.7	2–44	
Cohort B	20	13.4 ± 8.0	4–30	
Null	12	12.5 ± 8.3	4–36	
Null/Other	16	17.8 ± 11.8	5–44	
Other	14	18.9 ± 11.9	2–40	
Disease Duration (years)	42	18.4 ± 11.9	2–48	< 0.0001
Cohort A	22	11.6 ± 8.7	2–38	
Cohort B	20	25.9 ± 10.5	9–48	
Null	12	18.9 ± 8.3	8–33	ns
Null/Other	16	14.3 ± 11.1	2–34	
Other	14	22.6 ± 14.4	3–48	
Variant Classification				ns
Cohort A	22			
Null	4			
Null/Other	10			
Other	8			
Cohort B	20			
Null	8			
Null/Other	6			
Other	6			

The differences between Cohort A (able to perform 10 m in < 12 s) and B (unable or > 12 s), as well as the variant groups, are shown in Table 1. Twenty‐two of the subjects (52.4%) were assigned to Cohort A. This cohort had fewer patients with “null” variants (4 subjects (18.2%) compared to 8 (40%) in Cohort B) and a significantly shorter amount of time since the onset of symptoms, with an average disease duration of 11.6 years compared to 25.9 years in Cohort B (*p* < 0.001). Age of onset and the average amount of time since onset were relatively the same between the variant groups (disease duration unpaired *t*‐test *p* > 0.05). A complete list of individual variant classifications can be found in Appendix S1.

### 3.2 Test–Retest Reliability

**FIGURE 1:**
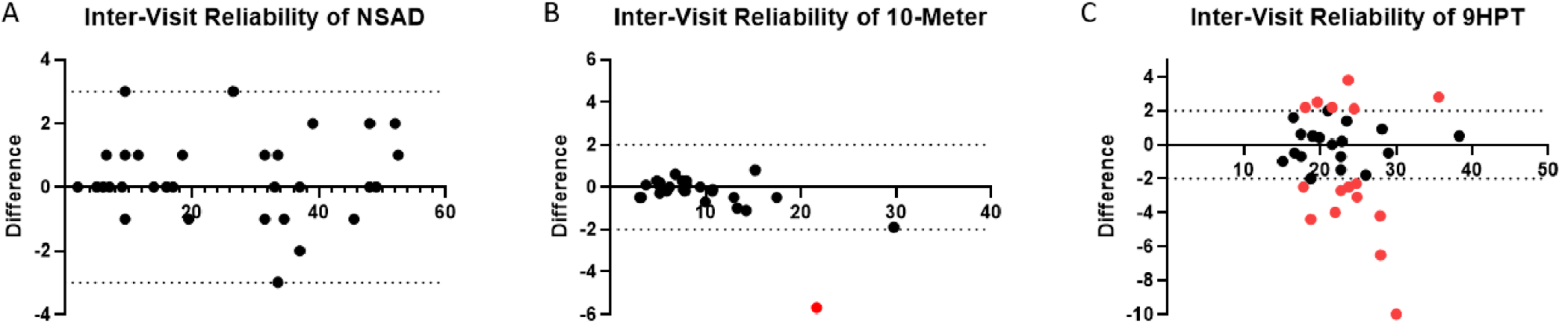
Inter‐visit reliability: Bland–Altman plots of variability in scoring between Baseline Day 1 and Baseline Day 2 of the NSAD (A), 10 m (B), and 9HPT (C) to evaluate inter‐visit reliability of individual COAs. Data points outside of allowed scoring ranges are considered unreliable. The NSAD (A) demonstrated random individual patient variability within the acceptable range while the 10 m (B) shows acceptable individual variability with a trend toward decreasing performance over the visits. The 9HPT (C) demonstrated significant individual variability despite high group reliability measured by ICC.

Test–retest reliability was analyzed for all in‐person baseline visits, excluding one subject who completed remote baseline assessments. All COAs demonstrated consistent group performance across both baseline visits, indicating high, statistically significant test–retest reliability of these functional COAs (ICC: 0.87–0.99, *p* < 0.001) (Table 2). Bland–Altman plots of the COAs demonstrate the individual variability in performance between the two testing days, with the 9HPT demonstrating significant individual variability across days despite high group reliability measured by ICC (Figure 1). Conversely, the NSAD demonstrates random individual patient variability within the acceptable range (Figure 1A) while the 10 m shows acceptable individual variability with a trend toward decreasing performance over the visits, which may indicate fatigue of repeated testing in those taking > 10 s to complete the test (Figure 1B).

**TABLE 2:** Inter‐visit reliability.

COA	*N*	Day 1	Day 2	Change	ICC
NSAD	40	24.6 ± 17.2 [2–52]	24.3 ± 17.1 [2–53]	0.3 ± 1.1 [−3–3]	0.99*
PUL	41	29.3 ± 8.1 [14–42]	29.1 ± 8.0 [16–42]	0.1 ± 1.6 [−4–5]	0.98*
10‐Meter (s)	26	9.7 ± 6.5 [3.3–30.7]	9.5 ± 5.8 [2.8–28.8]	−0.4 ± 1.2 [−5.7–0.8]	0.98*
Rise from Floor (s)	17	6.2 ± 5.4 [1.6–22.0]	6.5 ± 5.7 [1.3–18.9]	−0.03 ± 1.7 [−4.0–4.0]	0.95*
100‐Meter (s)	20	71.9 ± 29.7 [29.3–133.0]	74.0 ± 27.6 [29.8–126.1]	0.6 ± 12.5 [−24.5–48.0]	0.97*
TUG (s)	20	8.1 ± 3.7 [3.8–18.9]	8.4 ± 3.6 [3.5–17.8]	0.3 ± 0.9 [−1.1–3.2]	0.97*
4SC (s)	16	4.1 ± 2.5 [1.5–11.4]	3.9 ± 2.0 [1.2–9.5]	−0.3 ± 0.6 [−1.9–0.6]	0.97*
9HPT (s)	34	23.0 ± 5.7 [15.6–38]	22.1 ± 5.3 [14.6–38.5]	−0.8 ± 2.8 [−10.0–3.8]	0.86*

* *p* < 0.001.

### 3.3 Cross‐Sectional Performance of COA and PRO

All COAs and PROMs significantly correlated with each other (*r* = 0.40–0.87, *p* < 0.05), with the highest correlations of PROMs to the NSAD and PUL (*r* = 0.71–0.87, *p* < 0.05). At the time of baseline assessments, only 4 subjects (9.5%) had a total NSAD score < 4/54, with no subject scoring 0/54. Three additional subjects (7.1%) scored > 50 points, with no subject scoring 54/54, indicating a very minimal floor and ceiling effect for the LGMDR1 group. On the contrary, 8 subjects (19%) scored > 40/42 on the PUL, with 4 of those patients reaching a full score of 42/42, demonstrating a greater ceiling effect. No floor effect was observed as the lowest score was 14/42. The use of the 10 m proved effective, not only as a time cut‐off in dividing subjects into two distinct cohorts, but in identifying valuable time points of when subjects lost specific functional abilities. For instance, subjects with a 10 m time of > 11 s were found to have lost the ability to rise from the floor independently, and a time of > 15 s had lost the ability to rise from a chair, as seen in the performance of the NSAD.

### 3.4 Performance Between Cohorts

**FIGURE 2:**
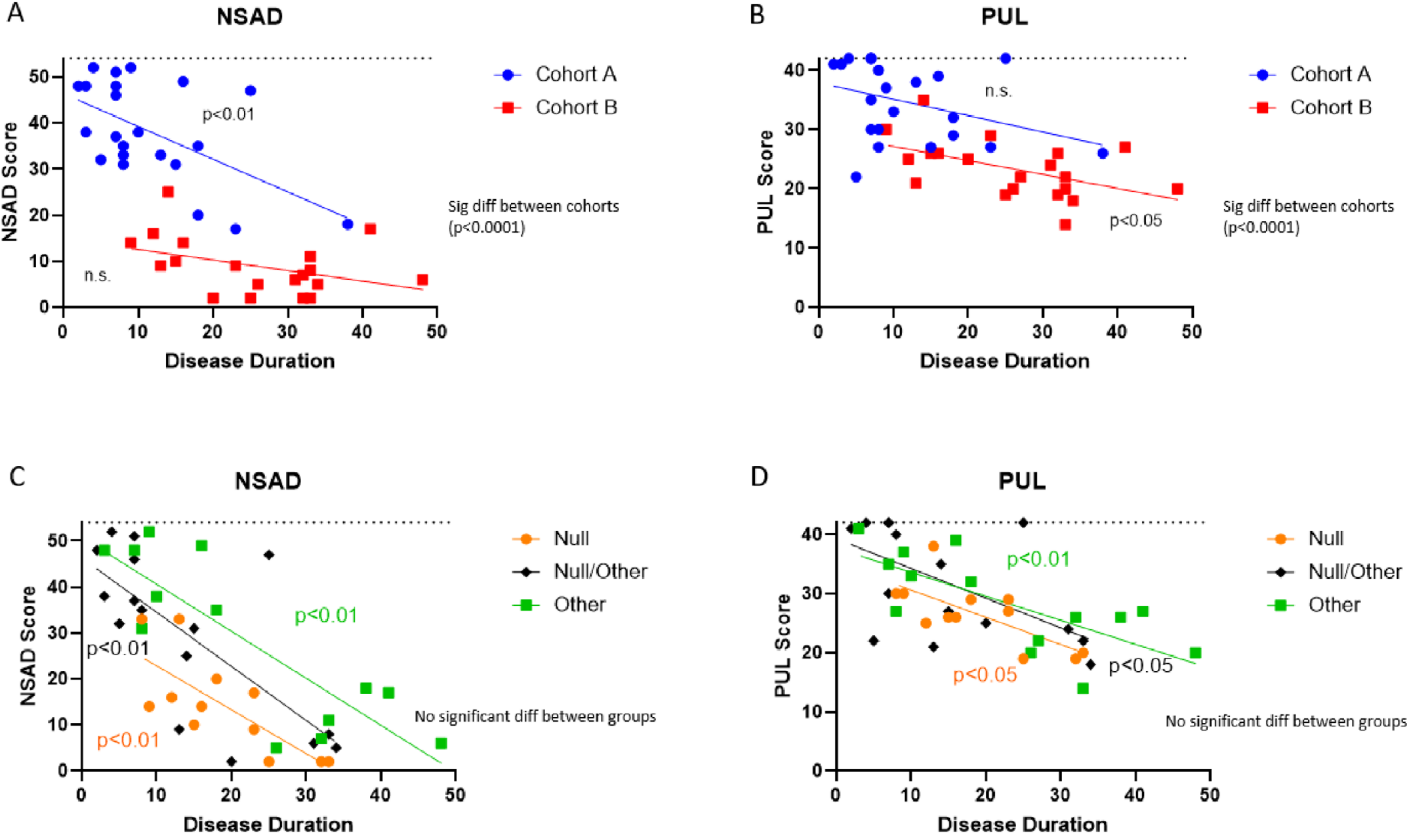
NSAD and PUL Comparison: Linear regression models of NSAD and PUL comparing performance between Cohort A and Cohort B (A, B) and between variant classification groups (C, D). Both the NSAD (A) and PUL (B) show a significant difference between Cohorts A and B, with higher scores seen in Cohort A indicating stronger functional abilities. There was no detectable difference in the performance of variant groups of the NSAD (C) or PUL (D).

Table 3 shows the average scores for each cohort, including standard deviation and range of scores. As expected, of the assessments completed in both cohorts, Cohort A demonstrated stronger functional abilities on the NSAD (Figure 2A; Mann–Whitney *p* < 0.001), PUL (Figure 2B; Mann–Whitney *p* < 0.001), 9HPT (*p* < 0.001), and Forced Vital Capacity (FVC) (*p* < 0.01). Only 6 subjects (30%) in Cohort B were able to ambulate 10 m without assistive devices, and none were able to rise from the floor, whereas 18 subjects (81.8%) in Cohort A were able to rise from the floor. A significant difference between the cohorts was also seen on all three PROMs (Mann–Whitney *p* < 0.001), with Cohort B self‐reporting greater limitations or inability to perform activities of daily living (ADL) due to decreased functional strength.

**TABLE 3:** COA comparisons.

	Cohort A	Cohort B	*p*
NSAD	38.1 ± 10.9 [17–52]	8.9 ± 6.1 [2–25]	< 0.001
PUL	34.7 ± 6.5 [22–42]	23.4 ± 4.9 [14–35]	< 0.001
10 M	7.0 ± 2.8 [3.3–13.8]	19.3 ± 6.9 [13.2–30.7]	< 0.001
9HPT	18.9 ± 2.2 [15.6–23.1]	26.5 ± 5.1 [17.9–38]	< 0.001
FVC	101.8 ± 25.7 [79–190]	75.0 ± 17.8 [41–107]	< 0.01

### 3.5 Performance Between Variants

Subjects in the “null” group had significantly slower times on the 100 m than the “null/other” group (*p* < 0.01). While there was no significant difference in the total performance of the NSAD (Figure 2C), only two subjects (16.7%) in the “null” group were able to rise from the floor independently and 7 subjects (58.3%) were able to ambulate 10 m without assistive devices at baseline compared to the “other” group (50% able to RFF and 64.3% ambulatory) and “null/other” groups (56.3% able to RFF and 68.8% ambulatory). The PUL score was not significantly different across the variant groups, with more homogeneity in the presentation of upper extremity involvement (Figure 2D). Unlike the cohort comparison, no significant difference between the variant groups was found with any of the PROMs.

## 4 Discussion

Our study is the largest sample to date to prospectively evaluate these specific functional COAs and PROs in patients with LGMDR1. We describe a cross‐sectional sample of baseline performance of standardized COAs and PROMs for the purpose of determining the validity and reliability of measures, such as the NSAD, to be used in patients with LGMDR1. Based on the results of our cohort, we have determined that this battery of performed assessments can be administered reliably across multiple international sites with trained physical therapists.

In our sample of patients with LGMDR1, the NSAD could continuously evaluate patients as they transitioned from ambulatory to non‐ambulatory, without reaching a floor or ceiling effect. Strong correlations with the other COAs and PROMs suggest the NSAD may eliminate the need for extensive testing due to the inclusivity of tasks with varying degrees of difficulty; however, we have insufficient data from the current analysis to measure change over time while the longitudinal data analysis is still pending. The use of one comprehensive assessment like the NSAD not only streamlines tracking of disease progression across international clinical sites, but also decreases the burden of extensive testing these individuals have historically completed [3]. Extensive testing is related to participant fatigue and may reduce retention in trials. Extensive testing may also limit the ability for sites in low resource countries to participate in clinical trials. While the NSAD proves to be a valid assessment on its own, combining this measure with other COAs like the 100 m or PUL can provide valuable insight into specific areas of interest, such as walking speed or upper extremity function in relation to a more global performance on the NSAD. Additionally, the 100 m could raise the ceiling effect in a trial of stronger patients while the PUL can lower the floor effect of the overall assessment into a non‐ambulatory cohort. The 10 m, which is also included as the last item of the NSAD, appears to be useful in predicting functional decline, which allows for valuable discussion between clinicians and patients regarding disease progression and plan of care in clinical settings. The 10 m time points could also be useful in establishing inclusion/exclusion criteria or for randomization in future clinical trials.

A previous natural history cohort study with 85 patients was genetically less heterogeneous, with 38 different CAPN3 variants and 57% of patients homozygous, all of whom had one of two different “null” variants [8]. Our cohort included 48 unique variants: 31 missense, 5 frameshift, 3 in‐frame deletions, 2 nonsense, and 7 expected to affect splicing (6 intronic, 1 synonymous). Thirty‐five participants were heterozygous for two different CAPN3 variants, and only seven (17%) were homozygous for a single variant. To enable comparison with the previous study, we also classified variants into two categories, “null” and “other,” based on their expected effects on the protein.

Despite a lack of statistical significance in performance on COAs between the variant groups, our study is suggestive of a trend of more functional limitations in the “null” group based on significantly slower 100 m times and higher reports of loss of ability to RFF. While the genotypic heterogeneity of our cohort may have contributed to the lack of a strong genotype–phenotype correlation, it is important to recognize that categorizing variants based on their predicted effects is imprecise. Some “null” variants may not result in an absent protein product, instead producing an altered protein with unknown functional consequence [22]. Similarly, while missense variants may not affect overall protein expression levels, they may still strongly disrupt normal protein function, though experimental demonstration of the extent to which an individual missense variant affects function is not commonly available [23]. Moreover, how residual calpain‐3 activity correlates with patient functional performance remains unclear. Further experimental characterization of the many pathogenic variants reported in CAPN3 is needed to fully understand the relationship between patient genotype and phenotype, particularly as patient muscle biopsies become less common and quantification of protein expression less accessible. The urgent need to develop a calpain‐3 activity assay is further supported by impending therapeutics that will need to establish the delivery of a functional calpain‐3 protein in LGMDR1 patients.

Our participants represent a wide range of age groups, with symptom onset as early as the first decade of life to as late as the fourth decade. Enrollment was limited in part due to a 20% total enrollment cap placed on Cohort B to allow for higher enrollment in Cohort A for longitudinal analysis. This restriction of non‐ambulatory patients or those transitioning to non‐ambulatory limits the ability of these study findings to be generalized to those on the weaker end of the disease spectrum. Furthermore, a delay in the enrollment of participants was largely due to the COVID‐19 pandemic, which limited the opening of several new sites in addition to the pausing of enrollment in existing sites.

To conclude, the GRASP‐LGMD consortium has identified a battery of COAs and PROs that are reliable and valid in evaluating functional abilities of patients with LGMDR1, in addition to quantifying functional differences based on age of symptom onset or variant classification. The COAs were tolerated well by our patients, as observed by an apparent lack of fatigue impacting performance of COAs at back‐to‐back visits or adverse events. Longitudinal analysis is necessary to evaluate the ability of these assessments to capture change in functional status over time, as well as improve our understanding of the meaningfulness of these changes to our patients and their families. Once subject visits are completed at all consortium sites, the GRASP‐LGMD consortium will begin this longitudinal analysis, including the potential application of these COAs and PROs in future clinical research trials.

## Author Contributions

N.E.J., C.C.W., L.N.A., L.P.L., J.M.S., M.W., S.D., T.M., K.D.M., P.B.K., M.K.J. contributed to protocol development and design of the study. All authors contributed to data collection. S.M.H. drafted a significant portion of the manuscript and figures. All authors contributed to review and approval of final manuscript.

## Conflicts of Interest

Dr. Hunn has received a travel award from Coalition to Cure Calpain 3 (C3). Dr. Alfano received consultant fees from Amicus Therapeutics and Asklepios Biopharmaceutical and receives funding via her institution and royalties from Sarepta Therapeutics. Dr. Lowes has joined Sarepta Therapeutics since the completion of this study. Dr. Iammarino serves as a consultant for ATOM international and receives salary support to her institution from Sarepta Therapeutics. Dr. Reash serves as a consultant for ATOM International and receives salary support to her institution from Sarepta Therapeutics. Dr. Statland has received grant funding from the NIH, MDA, CDC, FSHD Society, Friends of FSH Research, FSHD Canada, and ALSA; he is on the advisory board or serves as a consultant for Dyne, Avidity, Fulcrum, Roche, Kate, Sanofi, Epic Bio, ML Bio, Vertex, MireCule, and Merck. Dr. Dixon has served on advisory boards for Sarepta, Biogen, Genetech, Argenx, Alexion, Immunovant, and CSL Behring. Dr. Mozaffar has received research funding from ML Bio. He has consulted for Ask Bio. Dr. Laubscher receives support through her institution via funding from Edgewise Therapeutics, Italfarmaco, MLBio Solutions, Pfizer, Sarepta Therapeutics, and Virginia Commonwealth University. She is a paid consultant for ATOM International. Dr. Mockler receives funding from NIH grant 2 U54 NS053672‐11. She provides consultation services for ATOM International Limited, which includes consultation for Edgewise Therapeutics, Italfarmaco SpA, MLBio, NS Pharma, Pfizer, PTC Therapeutics Inc., Sarepta Therapeutics Inc., Capricor Therapeutics, AskBio, Biohaven, Dyne Therapeutics, PepGen, REGENXBIO Inc., Entrada Therapeutics, and Vertex Pharmaceuticals. Dr. Leung's institution has received research funding from Asklepios BioPharmaceutical Inc., Avidity Biosciences, Cumberland Pharmaceuticals, Edgewise Therapeutics, F. Hoffmann‐La Roche AG, Fulcrum Therapeutics, Harmony Biosciences, ML Bio Solutions Inc., Pfizer Inc., Sarepta Therapeutics, Seattle Children's Research Institute, the University of Kansas Medical Center Research Institute, and Virginia Commonwealth University. Dr. Kang has received research funding from Sarepta Therapeutics and ML Bio. He has served on advisory boards for ITF Therapeutics and Lupin Pharmaceuticals, and has consulted for Neurogene. Dr. James provides consultancy services for the following companies: ATOM International (covers consultancy services provided to Amicus Therapeutics Pty Ltd., Ascendis Pharma, Biomarin, Edgewise, Genethon, Italfarmaco, MLBio, NS Pharma, Pfizer, PTC Therapeutics, QED Therapeutics, Sarepta Therapeutics). Meredith has participated on Advisory Boards for F. Hoffman La Roche AG, Sarepta Therapeutics, and consultancies with Sarepta Therapeutics, Amicus, and Sanofi with fees paid to Newcastle University, and received fee support for PhD studies from the Jain Foundation. Dr. Clause has received consulting fees from Sarepta. Dr. Weihl has received funding and consulting fees from MLBio and Sarepta. Dr. Johnson has received research funds from Novartis, Takeda, PepGen, Sanofi Genzyme, Dyne, Vertex Pharmaceuticals, Fulcrum Therapeutics, AskBio, ML Bio, and Sarepta. He has provided consultation for Arthex, Angle Therapeutics, Juvena, Rgenta, PepGen, AMO Pharma, Takeda, Design, Dyne, AskBio, Avidity, and Vertex Pharmaceuticals. He has stock options from Myogene Therapies, Repeat RNA therapeutics, Angle therapeutics, and Juvena.

## Supporting information


Appendix S1.

## Data Availability

The data that support the findings of this study are available on request from the corresponding author. The data are not publicly available due to privacy or ethical restrictions.

## References

[1] S. Mohan , S. McNulty , C. Thaxton , et al., “Expert Panel Curation of 31 Genes in Relation to Limb Girdle Muscular Dystrophy,” Annals of Clinical Translational Neurology 11 (2024): 2268–2276. 39215466 10.1002/acn3.52127 PMC11537137

[2] R. Muni‐Lofra , E. Juanola‐Mayos , M. Schiava , et al., “Longitudinal Analysis of Respiratory Function of Different Types of Limb Girdle Muscular Dystrophies Reveals Independent Trajectories,” Neurology Genetics 9, no. 4 (2023): e200084. 37440793 10.1212/NXG.0000000000200084 PMC10335843

[3] M. B. Jacobs , M. K. James , L. P. Lowes , et al., “Assessing Dysferlinopathy Patients Over Three Years With a New Motor Scale,” Annals of Neurology 89, no. 5 (2021): 967–978. 33576057 10.1002/ana.26044

[4] A. G. Mayhew , M. K. James , U. Moore , et al., “Assessing the Relationship of Patient Reported Outcome Measures With Functional Status in Dysferlinopathy: A Rasch Analysis Approach,” Frontiers in Neurology 13 (2022): 828525. 35359643 10.3389/fneur.2022.828525 PMC8961025

[5] A. LoMauro , S. Gandossini , A. Russo , et al., “Over Three Decades of Natural History of Limb Girdle Muscular Dystrophy Type R1/2A and R2/2B: Mathematical Modelling of a Multifactorial Study,” Neuromuscular Disorders 31, no. 6 (2021): 489–497. 33836912 10.1016/j.nmd.2021.02.018

[6] I. F. Audhya , A. Cheung , S. M. Szabo , et al., “Progression to Loss of Ambulation Among Patients With Autosomal Recessive Limb‐Girdle Muscular Dystrophy: A Systematic Review,” Journal of Neuromuscular Diseases 9, no. 4 (2022): 477–492. 35527561 10.3233/JND-210771 PMC9398075

[7] A. P. Murphy , J. Morrow , J. R. Dahlqvist , et al., “Natural History of Limb Girdle Muscular Dystrophy R9 Over 6 Years: Searching for Trial Endpoints,” Annals of Clinical Translational Neurology 6, no. 6 (2019): 1033–1045. 31211167 10.1002/acn3.774 PMC6562036

[8] I. Richard , J. Y. Hogrel , D. Stockholm , et al., “Natural History of LGMD2A for Delineating Outcome Measures in Clinical Trials,” Annals of Clinical Translational Neurology 3, no. 4 (2016): 248–265. 27081656 10.1002/acn3.287 PMC4818744

[9] A. Doody , L. Alfano , J. Diaz‐Manera , et al., “Defining Clinical Endpoints in Limb Girdle Muscular Dystrophy: A GRASP‐LGMD Study,” BMC Neurology 24, no. 1 (2024): 96. 38491364 10.1186/s12883-024-03588-1 PMC10941356

[10] J. W. Sharpless , “Mossman's Problem‐Oriented Approach to Stroke Rehabilitation. The Nine‐Hole Peg Test of Finger Hand Coordination for the Hemiplegic Patient,” JAMA: The Journal of the American Medical Association 249, no. 13: 1773 (1983).

[11] D. Podsiadlo and S. Richardson , “The Timed “Up & Go”: A Test of Basic Functional Mobility for Frail Elderly Persons,” Journal of the American Geriatrics Society 39, no. 2 (1991): 142–148. 1991946 10.1111/j.1532-5415.1991.tb01616.x

[12] M. H. Brooke , G. M. Fenichel , R. C. Griggs , et al., “Clinical Investigation in Duchenne Dystrophy: 2. Determination of the “Power” of Therapeutic Trials Based on the Natural History,” Muscle & Nerve 6, no. 2 (1983): 91–103. 6343858 10.1002/mus.880060204

[13] A. G. Mayhew , G. Coratti , E. S. Mazzone , et al., “Performance of Upper Limb Module for Duchenne Muscular Dystrophy,” Developmental Medicine and Child Neurology 62, no. 5 (2020): 633–639. 31538331 10.1111/dmcn.14361

[14] M. Pane , E. S. Mazzone , L. Fanelli , et al., “Reliability of the Performance of Upper Limb Assessment in Duchenne Muscular Dystrophy,” Neuromuscular Disorders 24, no. 3 (2014): 201–206. 24440357 10.1016/j.nmd.2013.11.014

[15] L. N. Alfano , N. F. Miller , K. M. Berry , et al., “The 100‐m Timed Test: Normative Data in Healthy Males and Comparative Pilot Outcome Data for Use in Duchenne Muscular Dystrophy Clinical Trials,” Neuromuscular Disorders 27, no. 5 (2017): 452–457. 28279570 10.1016/j.nmd.2017.02.007

[16] M. K. James , L. N. Alfano , R. Muni‐Lofra , et al., “Validation of the North Star Assessment for Limb‐Girdle Type Muscular Dystrophies,” Physical Therapy 102, no. 10: 113 (2022). 10.1093/ptj/pzac113 PMC9586158 35932452

[17] P. L. Hudak , P. C. Amadio , and C. Bombardier , “Development of an Upper Extremity Outcome Measure: The DASH (Disabilities of the Arm, Shoulder and Hand) [Corrected]. The Upper Extremity Collaborative Group (UECG),” American Journal of Industrial Medicine 29, no. 6 (1996): 602–608. 8773720 10.1002/(SICI)1097-0274(199606)29:6<602::AID-AJIM4>3.0.CO;2-L

[18] L. Van der Velde , P. Y. Van den Bergh , N. Goemans , and J. L. Thonnard , “ACTIVLIM: A Rasch‐Built Measure of Activity Limitations in Children and Adults With Neuromuscular Disorders,” Neuromuscular Disorders 17, no. 6 (2007): 459–469, 10.1016/j.nmd.2007.02.013. 17433675

[19] W. T. Riley , N. Rothrock , B. Bruce , et al., “Patient‐Reported Outcomes Measurement Information System (PROMIS) Domain Names and Definitions Revisions: Further Evaluation of Content Validity in IRT‐Derived Item Banks,” Quality of Life Research 19, no. 9 (2010): 1311–1321. 20593306 10.1007/s11136-010-9694-5 PMC3670674

[20] D. Cella , W. Riley , A. Stone , et al., “The Patient‐Reported Outcomes Measurement Information System (PROMIS) Developed and Tested Its First Wave of Adult Self‐Reported Health Outcome Item Banks: 2005–2008,” Journal of Clinical Epidemiology 63, no. 11 (2010): 1179–1194. 20685078 10.1016/j.jclinepi.2010.04.011 PMC2965562

[21] A. C. Nascimbeni , M. Fanin , E. Tasca , and C. Angelini , “Transcriptional and Translational Effects of Intronic CAPN3 Gene Mutations,” Human Mutation 31, no. 9 (2010): E1658–E1669. 20635405 10.1002/humu.21320 PMC2966865

[22] M. C. Dyle , D. Kolakada , M. A. Cortazar , and S. Jagannathan , “How to Get Away With Nonsense: Mechanisms and Consequences of Escape From Nonsense‐Mediated RNA Decay,” Wiley Interdisciplinary Reviews 11, no. 1 (2020): e1560. 31359616 10.1002/wrna.1560 PMC10685860

[23] M. Fanin , A. C. Nascimbeni , and C. Angelini , “Screening of Calpain‐3 Autolytic Activity in LGMD Muscle: A Functional Map of CAPN3 Gene Mutations,” Journal of Medical Genetics 44, no. 1 (2007): 38–43. 16971480 10.1136/jmg.2006.044859 PMC2597906

