# Supplementary material for: Clinical Trial Readiness in Limb Girdle Muscular Dystrophy R1 (LGMDR1): A GRASP Consortium Study
Source: Ann Clin Transl Neurol. 2025 Apr 16;12(6):1179–86. doi: 10.1002/acn3.70049 (PMC12172116; doi:10.1002/acn3.70049)
Supplement: Supplementary file 1 — Appendix S1. [file ACN3-12-1179-s001.pdf]

Participant ID	Variant 1			Variant 2			Genotype Class
	Description	ClinVar/CA ID	Pathogenicity Classification	Description	ClinVar/CA ID	Pathogenicity Classification	
1	c.633G>C p.(Lys211Asn)	284946	VUS; Likely Pathogenic	c.1505T>C p.(Thr501Ile)	282512	VUS; Likely Pathogenic	Other
2	c.1981delA p.(Gln660_Ile661insTer)	194691	Pathogenic	c.598_612del p.(Phe200_Leu204del)	166786	Pathogenic	Other/Null
3	c.550delA p.(Thr184ArgfsTer36)	17621	Pathogenic	homozygous	homozygous	homozygous	Null
4	c.1469G>A p.(Arg490Gln)	17622	Pathogenic	c.2148G>T p.(Glu716Asp)	285340	Pathogenic	Other
5	c.550delA p.(Thr184ArgfsTer36)	17621	Pathogenic	c.1250C>T p.(Thr417Met)	281505	Pathogenic	Other/Null
6	c.1468C>T p.(Arg490Trp)	166790	Pathogenic	c.1981delA p.(Gln660_Ile661insTer)	194691	Pathogenic	Other/Null
7	c.2362_2363delinsTCATCT p.(Arg788SerfsTer14)	17618	Pathogenic	c.1782G>A p.(Val594=)	2738034	Pathogenic	Null
8	c.1194-9A>G	217146	Pathogenic	c.1187A>G p.(Glu396Gly)	497565	VUS; Likely Pathogenic	Other/Null
9	c.2306G>A p.(Arg769Gln)	17613	Pathogenic	c.598_612del p.(Phe200_Leu204del)	166786	Pathogenic	Other
10	c.1714C>T p.(Arg572Trp)	217152	Pathogenic	c.2242C>T p.(Arg748Ter)	283259	Pathogenic	Other/Null
11	c.598_612del p.(Phe200_Leu204del)	166786	Pathogenic	c.1354G>T p.(Asp452Tyr)	166789	VUS; VUS	Other
12	c.1435A>G p.(Ser479Gly)	92405	Pathogenic	seq[GRCh37]del(15)(15q15.1), NC_000015.9:g.42651696_42652315del (exon 1 deletion)	CA2837589099	Pathogenic	Other/Null
13	c.1465C>T p.(Arg489Trp)	217150	Pathogenic	c.1715G>A p.(Arg572Gln)	17614	Pathogenic	Other
14	c.146G>A p.(Arg49His)	217151	Pathogenic	c.865C>T p.(Arg289Trp)	282646	Likely Pathogenic	Other
15	c.550delA p.(Thr184ArgfsTer36)	17621	Pathogenic	homozygous	homozygous	homozygous	Null
16	c.2362_2363delinsTCATCT p.(Arg788SerfsTer14)	17618	Pathogenic	c.2393C>A p.(Ala798Glu)	92414	Pathogenic	Other/Null
17	c.550delA p.(Thr184ArgfsTer36)	17621	Pathogenic	homozygous	homozygous	homozygous	Null
18	c.2120A>G p.(Asp707Gly)	468648	Pathogenic	c.1194-2del	2758089	Pathogenic	Other/Null
19	c.1319G>A p.(Arg440Gln)	217147	Pathogenic	c.1981delA p.(Gln660_Ile661insTer)	194691	Pathogenic	Other/Null
20	c.550delA p.(Thr184ArgfsTer36)	17621	Pathogenic	c.1303G>A p.(Glu435Lys)	282173	Pathogenic	Other/Null
21	c.1327T>C p.(Ser443Pro)	662490	VUS; Likely Pathogenic	c.1746-20C>G	92408	VUS; Pathogenic	Other
22	c.620A>C p.(Lys207Thr)	285460	VUS; Likely Pathogenic	c.1746-20C>G	92408	VUS; Pathogenic	Other
23	c.1327T>C p.(Ser443Pro)	662490	Pathogenic	c.1746-20C>G	92408	VUS; Pathogenic	Other
24	c.550delA p.(Thr184ArgfsTer36)	17621	Pathogenic	c.380-18_380-3del	285927	VUS; VUS	Null
25	c.759_761delGAA p.(Lys254del)	197624	Pathogenic	c.1256A>G p.(Asp419Gly)	284518	VUS; Likely Pathogenic	Other
26	c.1076C>T p.(Pro359Leu)	198690	VUS; Likely Pathogenic	c.1256A>G p.(Asp419Gly)	284518	VUS; Likely Pathogenic	Other
27	c.2290del p.(Asp764ThrfsTer12)	497201	Pathogenic	c.2362_2363delinsTCA p.(Arg788SerfsTer13)	CA2695218823	Pathogenic	Null
28	c.1993-1G>A	282494	Pathogenic	c.2105C>T p.(Ala702Val)	283099	Pathogenic	Other/Null
29	c.1342C>T p.(Arg448Cys)	280038	Pathogenic	c.1115+5G>C	265521	Pathogenic	Other/Null
30	c.1468C>T p.(Arg490Trp)	166790	Pathogenic	c.550delA p.(Thr184ArgfsTer36)	17621	Pathogenic	Other/Null
31	c.1333G>A p.(Gly445Arg)	284515	Pathogenic	c.146G>A p.(Arg49His)	217151	Pathogenic	Other
32	c.550delA p.(Thr184ArgfsTer36)	17621	Pathogenic	homozygous	homozygous	homozygous	Null
33	c.145C>T p.(Arg49Cys)	193037	Pathogenic	c.2243G>A p.(Arg748Gln)	128570	Pathogenic	Other
34	c.759_761delGAA p.(Lys254del)	197624	Pathogenic	c.2134C>T p.(Leu712Phe)	195450	Pathogenic	Other
35	c.550delA p.(Thr184ArgfsTer36)	17621	Pathogenic	c.2288A>G p.(Tyr763Cys)	282681	Pathogenic	Other/Null
36	c.550delA p.(Thr184ArgfsTer36)	17621	Pathogenic	c.1435A>G p.(Ser479Gly)	92405	Pathogenic	Other/Null
37	c.550delA p.(Thr184ArgfsTer36)	17621	Pathogenic	c.1622G>A p.(Arg541Gln)	92407	Pathogenic	Other/Null
38	c.550delA p.(Thr184ArgfsTer36)	17621	Pathogenic	homozygous	homozygous	homozygous	Null
39	c.550delA p.(Thr184ArgfsTer36)	17621	Pathogenic	c.1194-9A>G	217146	Pathogenic	Null
40	c.550delA p.(Thr184ArgfsTer36)	17621	Pathogenic	c.1194-9A>G	217146	Pathogenic	Null
41	c.717del p.(Phe239LeufsTer14)	284807	Pathogenic	homozygous	homozygous	homozygous	Null
42	c.717del p.(Phe239LeufsTer14)	284807	Pathogenic	homozygous	homozygous	homozygous	Null
